# Surface Profile Studies of Photoinduced Gratings Made with DCG Films with Optional Papain Development

**DOI:** 10.3390/gels8020102

**Published:** 2022-02-09

**Authors:** Sergio Calixto, Valeria Piazza, Guillermo Garnica

**Affiliations:** Centro de Investigaciones en Optica, Loma del Bosque 115, León 37150, Mexico; vpiazza@cio.mx (V.P.); garnica@cio.mx (G.G.)

**Keywords:** dichromated gelatin films, papain, surface relief, diffraction gratings

## Abstract

The use of surface relief structures is increasing in the field of optics. A study of photoinduced relief using dichromated gelatin films with different thickness is described in this paper. Two light sources were used: a laser (λ = 468 nm) and an ultraviolet mercury-metal halide lamp. Gratings with low spatial frequencies were contact-copied on the DCG (dichromated gelatin) films. Two development processes were used, one included washing the plates with just water and the other with a mixture of water and papain. This enzyme is used to improve the gratings’ relief which was studied with a profilometer. For the development process with just water, it was found that when gratings were recorded using visible or UV light, the height profile inversely correlated to spatial frequencies. For short exposure times, the reliefs showed a sinusoidal profile. When visible light was used, the DCG areas where the Ronchi grating had transparent slits showed a flat relief and the areas where the Ronchi grating had opaque slits showed a round peak, with the peak being taller than the flat surface. In contrast, when UV light was used, the flat surfaces were taller than the peaks. The relief height increased up to seven times when papain was used.

## 1. Introduction

Since the 1800s, dry films of chromates and dichromates mixed with organic materials such as gelatin, fish glue, gum arabic, or shellac were known to be affected by light [1]. The films become hard and less soluble in areas exposed to radiation. So that when washed with an appropriate solvent, only those areas which were exposed to light through a negative remain in the supporting material, forming a relief image. This process has been used in the printing industry, where one film could be used for 2000 impressions.

In 1968, Shankoff [2,3] proposed the use of dichromated gelatin plates (DCG plates) as the material to record holograms. He found diffraction efficiencies of 33% for thin phase gratings and 95% for thick holograms. The information is stored in the gelatin bulk as changes in refractive index. DCG plates can be made with gelatin and dichromates or with Kodak 649 F plates used as gelatin films where the silver halide has been removed with the development and fixing process.

Besides DCG plates, other materials have been used to fabricate optical relief elements, such as albumen [4], acrylamide dry films [5], polyacrylamide wet films (used to record transient interference patterns) [6], silicone [7,8], gelatin [9,10], liquid resins [11], and silver halide plates [12,13,14,15,16,17,18]. The relief elements comprise among others diffraction gratings, lenses, zone plates, holograms, and computer-generated holograms (CGH). Recently the relief elements were applied as host in the alignment of liquid crystals (LC) for applications such as photoswitchable gratings [19,20], birefringence gratings [21,22,23], beamsplitters [24] and in polarizing gratings [25]. 

Chemicals such as photoresists are commonly deployed to fabricate relief optical elements, but their use poses a threat for the environment and for the users. Gelatin is a good candidate for a safer fabrication of surface relief optical elements; therefore, there is a need to study and characterize the formation of the relief structures with gelatin films.

Gelatin is colorless, odorless, tasteless, non-soluble in organic solvents, environmentally friendly, easy to handle and to purchase, and cost effective, in addition it displays good clarity, biocompatibility, and biodegradability, among other characteristics. Moreover, the properties of gelatin such as its viscosity, gel strength, softening behavior, thixotropy, and melting point can be modified by UV light, heat, chemicals, and ultrasound. 

In optics, when a photosensitive material is characterized, the recording of sinusoidal patterns showing different spatial frequencies is usually performed. In the case of relief elements, the depth and the form of the surface relief depends on the exposure time. The analysis of these aspects was partially performed and reported in references [12,13,14,15,16,17,18], using DCG films. In these works, spatial frequencies between 60 and 200 L/mm are used, and the reliefs generated are approximately 0.4 µm to 1.4 µm thick, with a development step with water and a postprocessing method with UV light. These studies are mainly focused on characterizing the height of the reliefs, but not the shape. Unfortunately, the reliefs found are low for some applications, thus, it is of interest to develop methods to increase the height of the relief and the recording with low spatial frequencies. Besides the aforementioned works, another one [26] mentions the use of an enzyme, papain, for the development process of DCG films. This work does not analyze the height and form of the profiles of the recorded gratings or the action of exposure time.

In the present article we provide a study of the surface profile of DCG films when Ronchi gratings were contact-copied on the DCG surface. Spatial frequency, exposure time, DCG film thickness, and wavelength of recording light were the variables at exposure time, with a following development step performed either with water or a papain solution. Then, the form and depth of the reliefs were studied with a profilometer.

The information has been organized as follows. In Section 2, the general characteristics of gelatin are shown. In addition, a discussion of the efforts of other authors to increase the profile of DCG plates that recorded interference patterns [14,15,16,17,18] and the use of acrylamide material [5] as a substitute for DCG in the fabrication of relief elements is undertaken. Section 3 describes film preparation, the development process, film transmittance, grating recordings with laser and UV light, the use of papain in the development process, and the display of diffracted orders. Section 4 provides a discussion of the results.

## 2. Theory

Gelatin is a natural product made by the hydrolysis of collagen [27,28]. The source of collagen is normally either hide or bone that has been extracted with acid to remove minerals. The hydrolysis may be conducted either in basic solution or acid. The purity of gelatin depends on the source of collagen and its treatment prior to hydrolysis. Gelatin is a protein, and its large molecules are synthesized in nature from many molecules of amino acids.

The swelling of gelatin layers is of major importance in processing emulsion coatings. The coating is deposited over a film base, polymer, or glass, and upon drying heavy stress is generated at the interface between the emulsion and support [29]. This stress can, in extreme conditions, strip the emulsion from the support or cause so much swelling at the surface of the coating, compared with the stress that the film suffers near the base, that it results in a pattern of ridges and valleys, a condition called reticulation. Even the support, polymer, or glass, can bend.

The gelatin film can be modified by chemical reactions that introduce permanent cross-links between gelatin chains, a process called hardening. Aluminum and chromium are chemical hardeners. Another method to harden the gelatin films is by UV light that likely involves a temporary rupture of certain bonds, resulting in a rearrangement to a more stable structure [27].

The sensitivity of DCG films spans from approximately 330 nm to 550 nm [2]. In holography, widely used lasers for the recording of interference patterns with DCG films are the Argon laser typically emitting at 514 nm and 488 nm, the He-Cd laser emitting at 440 nm and 325 nm, and the Diode-pumped solid-state laser (DPSSL, Neodymium-doped YAG laser) emitting at 468 nm and 532 nm. With these wavelengths, crosslinking among the gelatin chains is obtained. The modulations can be present as bulk refractive index or surface profile changes. One example of UV recording was performed with a He-Cd laser emitting at 325 nm [30]. The authors used DCG plates that were fabricated similarly to 649 F plates. Diffraction gratings with a spacing of 0.5 microns were recorded and presented diffraction efficiencies of about 90% when red light (632.8 nm) was used. 

Efforts to increase the surface relief of silver halide and DCG plates using short-wave UV light in the development process were made, as reported in references [14,15,16,17,18]. At recording time, a 440 nm He-Cd laser light was used as the light source. A double beam sinusoidal interference pattern was recorded by the plates. The spatial frequency of the pattern varied between 60 lines/mm and 220 lines/mm. The development plates process included: (a) dipping the plates in a 20% sodium sulfate solution in water for 5 min, (b) washing in running water for 20 min and drying at room temperature, (c) irradiating the dried layers with a quartz mercury lamp for 40 min, (d) washing in water for 5 min, and finally (e) drying at room temperature. Reliefs of 0.4 µm to 0.6 µm, measured with a MII-4 micro-interferometer, were reported. Besides the use of the mercury lamp in the development process, an Excimer lamp and a Xe + Cl_2_ lamp were also used. The Mercury lamp had a radiation power of 15 W with a spectral region between λ = 240 nm and 320 nm, the Excimer lamp had a power of 10 W, with a spectral region of λ = 230 nm–320 nm, and the Xe + Cl_2_ lamp a radiation power of 10 W in the spectral region of λ =197 nm–230 nm. DCG layers with thicknesses ranging between 14 µm and 90 µm were made.

The mechanism that generated the relief, first suggested in reference [29] and later mentioned by authors [14,15,16,17,18], is the following: when the sinusoidal interference pattern generated with light having wavelengths between 320 and 550 nm illuminates the DCG plate, the physicochemical properties of gelatin are changed by selective tanning (cross-linking bonds) in the areas where the interference pattern crests show high intensity. These highly exposed parts should be less subject to the destructive effect of short-wave UV radiation (λ ≤ 250 nm–270 nm) during the development step, consequently, irradiation with short-wave UV light should give a pronounced surface relief when the plates are developed in water. The subsequent processing with water leads to the removal of photodegraded gelatin where the degree of tanning was lower. Surface reliefs were found in the range from 0.15 microns, when the plates did not undergo UV illumination, to 1.4 microns when the short-wave UV process was applied. Diffraction efficiencies of gratings that underwent UV process were approximately 25% when light had a λ = 632.8 nm.

Another effort to increase the depth relief of DCG plates was reported in reference [26]. In this study a mixture of papain and water was used. Papain [31] is an enzyme of high research interest mainly in the food industry and forage. It exhibits broad proteolytic activity, can work in a rather flexible range of temperatures, and its activation can be modulated by the use of its principal activator, cysteine, by immobilization, or both [32]. This study [26] only mentioned some preliminary experiments and applications, suggesting that additional work must be conducted in order to characterize the reliefs of the recorded gratings.

A different approach to make relief elements was presented in reference [5]. These authors used acrylamide films with a recording wavelength of 532 nm, finding that as spatial frequency of the interference sinusoidal pattern increased, the surface relief decreased. An investigation of the dependence of the photoinduced surface relief profile as a function of recording intensity, UV post exposure, thickness of the sample, composition of the polymer, and temperature was undertaken with the help of an interferometer that measured the depth profile. Non-sinusoidal surface relief gratings profiles, depending on the sample thickness, were observed at low spatial frequencies.

## 3. Experimental

### 3.1. Film Sample Preparation

In the present work, the fabrication of gelatin films was performed with a commercial product (Gelatin, Sigma, Type B, G9382, CAS: 9000-70-8, 225 bloom, Sigma-Aldrich, St. Louise, MO, USA). Gelatin films were fabricated with the following method. The substrates were 10 cm × 5 cm glass plates with 2 mm thickness. To make the gelatin thin films, a mixture of gelatin, 1.25 g, and water, 40 mL, was prepared. It was magnetically stirred and heated to 50 °C. Later, several milliliters were poured over the leveled plates. The amount of the mixture depended on the desired film thickness. For example, to make 20 microns thick gelatin layers, 2 milliliters were used. After the mixture dried, the gelatin films were immersed in a 2% w/v ammonium dichromate (J.T. Baker, 0688-1, PM252, Avantar, Radnor, Philadelphia, PA, USA) solution in distilled water for 5 min. Then they were left to dry overnight. Before the exposure to light, the plates were cut into four 5 cm × 2.5 cm pieces.

### 3.2. Films Development

When DCG plates are exposed to light, cross-linking (hardening) is established among the gelatin chains. The development process consisted of two methods: (a) only a rinsing step, in which the plates were rinsed in water for 10 min, followed by drying, or (b) after the water rinsing step, the plates were immersed in a mixture of papain (Sigma, P-3375, Sigma-Aldrich, St. Louise, MO, USA) and water. A total of 1.2 g of papain was dissolved in 40 mL of water and placed in the magnetic stirrer for 1 h. The grating was immersed in the papain mixture for 10 min. Plates were left to dry overnight prior to use. 

When the DCG films are placed in water, they swell. This swelling will be more pronounced in those regions where no light crosslinked the chains. At drying time, due to the heavy stress between the illuminated and non-illuminated areas, a relief is created. The activity of the papain solution on gelatin consists of the breaking of the crosslinks among gelatin chains. This is more pronounced in the non-illuminated areas. In the drying step, great forces within the film are present. The areas where there is a crosslink exert a powerful force over the uncrosslinked areas. Possibly, the transfer of material takes place between the uncrosslinked and crosslinked parts. Thus, when the films dry, a surface modulation will be present.

### 3.3. Gelatin Films Transmittance

**Figure 1:**
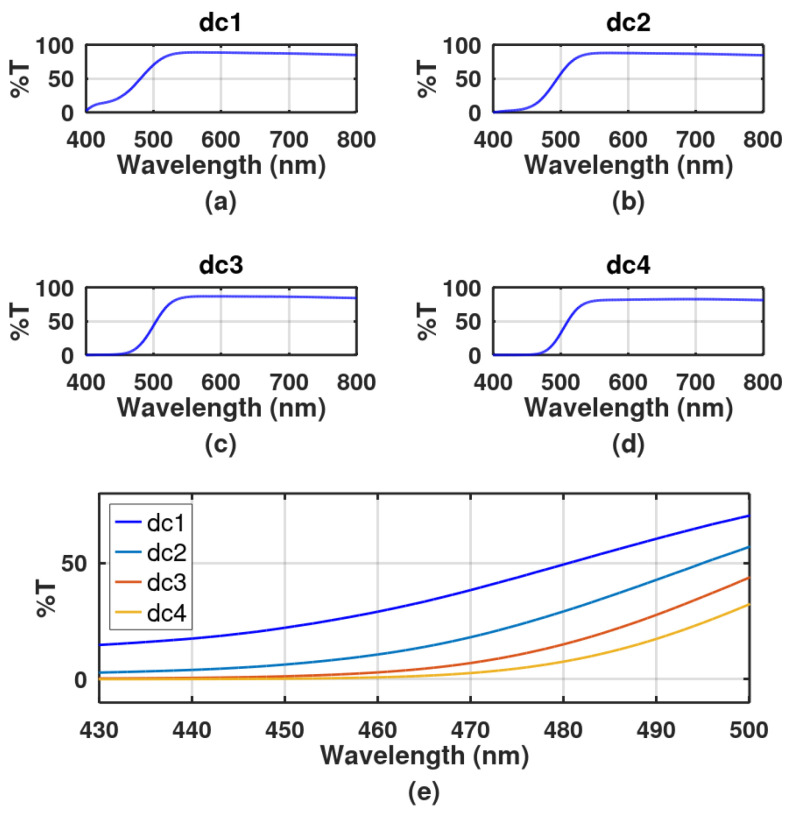
Transmittance as a function of wavelength for DCG plates soaked in water (50 mL) with different dichromate amounts, (**a**) 0.5 g, (**b**) 1 g, (**c**) 1.5 g, and (**d**) 2 g. DCG plates’ thickness was 30 µm. (**e**) Enlarged section between 430 nm and 500 nm showing the transmittance for the four concentrations used: dc 1, 10 g/L, dc 2, 20 g/L, dc 3, 30 g/L, and dc 4, 40 g/L.

The transmittance of the DCG plates was studied with a spectrometer (Agilent Technologies, UV-Vis-NIR, Carey 5000, Agilent technologies, Santa Clara, CA, USA). Four plates with the same thickness, 30 µm, were studied. They were immersed in four different solutions that contained 10 g/l 20 g/L, 30 g/L, and 40 g/L of dichromate. Results are presented in Figure 1. Since blue light with a wavelength of 468 nm was highly absorbed, in the following experiments this wavelength was used.

### 3.4. Exposure to Light

**Figure 2:**
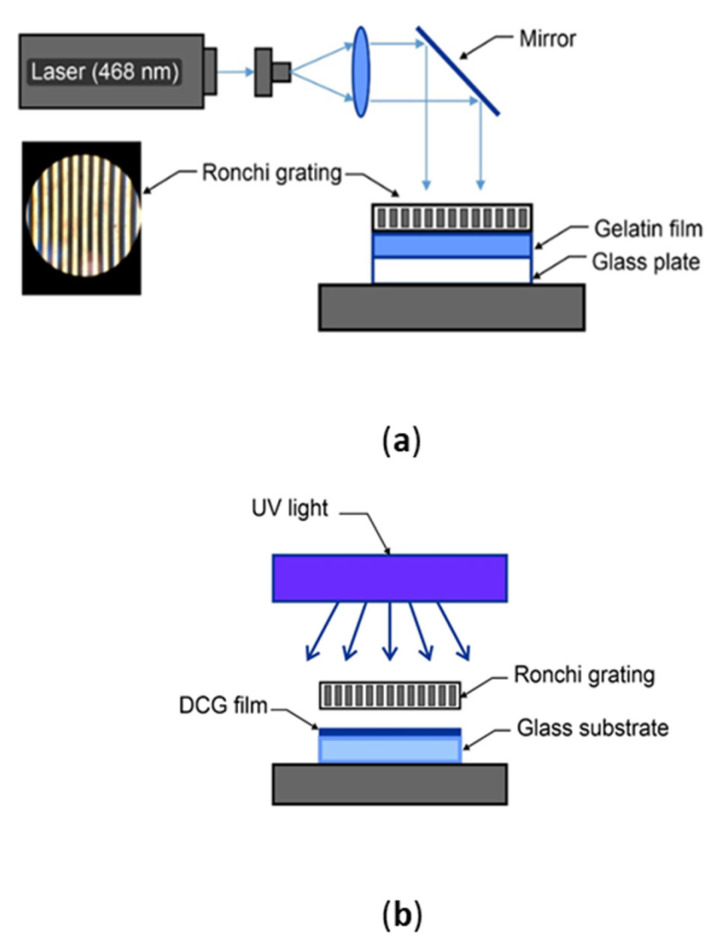
(**a**) Optical configuration using a laser for the DCG films exposure. Ronchi grating consisted of dark and transparent slits. (**b**) Optical configuration using an extended UV light for the DCG films exposure.

The optical configurations used are shown in Figure 2. Light sources were a Diode Pumped Solid State Laser (DPSSL, Neodymium-doped YAG laser, Edmund, Strafford County, NH, USA) giving 468 nm light, as shown in Figure 2a, or a high-pressure UV lamp (Loctite Zeta 7411, model 98027, UV flood curing system, mercury metal halide 400 Watt, Henkel, Hartford County, CT, USA) [33], as shown in Figure 2b. During the exposure, Ronchi gratings were placed over the DCG plates. Ronchi rulings were 2 cm × 2 cm. The following spatial frequencies were used: 4 L/mm (line width 250 microns), 6 L/mm (line width 166 microns), and 10 L/mm (line width 100 microns). 

**Figure 3:**
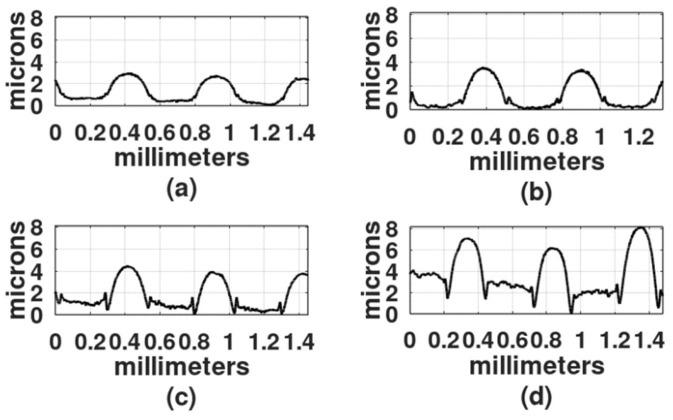
Profiles of gratings made with a 4 L/mm Ronchi grating. DCG film thickness was 30 µm. Exposure times: (**a**) 2 min, (**b**) 5 min, (**c**) 9 min, and (**d**) 18 min.

**Figure 4:**
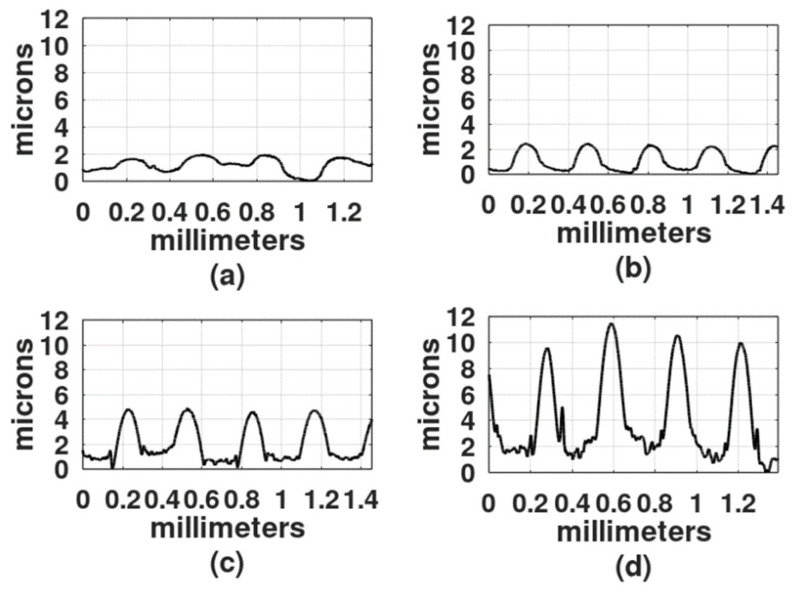
Profiles of gratings made with a 6 L/mm Ronchi grating. DCG film thickness was 30 µm. Exposure times: (**a**) 2 min, (**b**) 5 min, (**c**) 9 min, and (**d**) 18 min.

**Figure 5:**
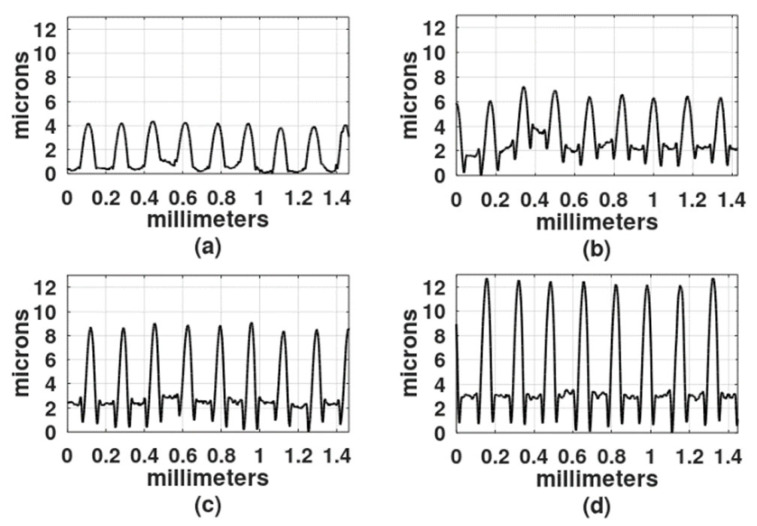
Profiles of gratings made with a 10 L/mm Ronchi grating. DCG film thickness was 30 µm. Exposure times: (**a**) 2 min, (**b**) 5 min, (**c**) 9 min, and (**d**) 18 min.

The light exposure parameters were: Ronchi ruling spatial frequency, light intensity, exposure time, wavelength of the light source, and gelatin film thickness. In the first study, the film thickness was 30 microns, the light intensity was 4 mw/cm^2^, and because DCG is more sensitive in the blue region of the spectrum than in the green region [2], the first recordings were made with a DPSS laser (468 nm). To select the exposure times, a series of exposures were performed from 30 s to 25 min. Gratings obtained with exposure times shorter than 2 min showed a very shallow relief. On the other hand, gratings made with exposure times longer than 20 min showed a central dark spot with gelatin presenting reticulation or cracks. Thus, the selected exposure times for each spatial frequency were: 2 min, 5 min, 9 min, and 18 min. Figure 3, Figure 4 and Figure 5 show the results when the film surface profile was studied with a surface analyzer (Federal Products Co., Providence, RI, USA, model Surfanalyzer 4000).

In Figure 3, Figure 4 and Figure 5 it can be appreciated that when exposure times were short, for example, 2 min or 5 min, the plots showed a sinusoidal behavior. However, when the exposure times increased, the height of the crests, where the Ronchi grating had transparent slits, began to increase and the valleys, where the Ronchi grating had black slits, displayed a rather horizontal surface. 

**Figure 6:**
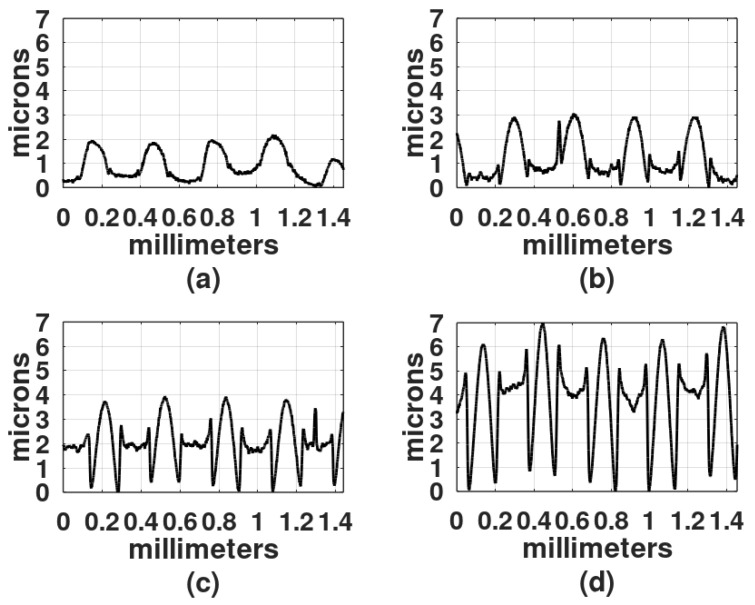
Profiles of gratings made with a 6 L/mm Ronchi grating. DCG film thickness was 50 µm. Exposure times: (**a**) 2 min, (**b**) 5 min, (**c**) 9 min, and (**d**) 18 min.

**Figure 7:**
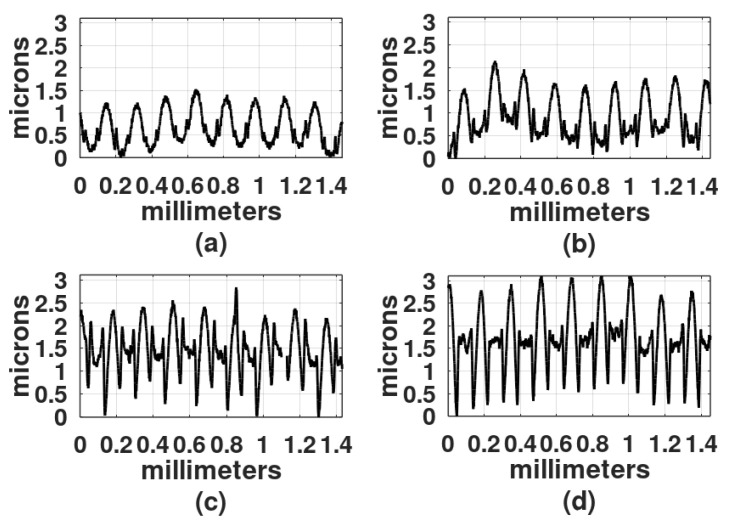
Profiles of gratings made with a 10 L/mm Ronchi grating. DCG film thickness was 50 µm. Exposure times: (**a**) 2 min, (**b**) 5 min, (**c**) 9 min, and (**d**) 18 min.

Besides the film thickness of 30 µm another film thickness was studied, 50 µm. Two spatial frequencies were used: 6 L/mm and 10 L/m. Results are shown in Figure 6 and Figure 7. 

In Figure 6d and Figure 7d the height difference between the crests and valleys is not as large as in the plots in Figure 3, Figure 4 and Figure 5. 

**Figure 8:**
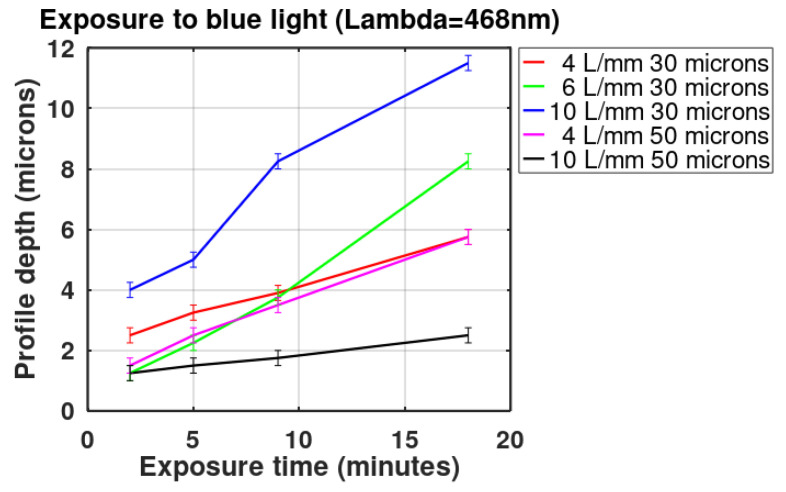
Gratings’ highest profile depth as a function of exposure times. Parameters are the spatial frequency and the DCG film thickness.

The depth of three of the highest reliefs, for each plot in Figure 3, Figure 4, Figure 5, Figure 6 and Figure 7 was measured, and the mean was plotted; the results are shown in Figure 8. The highest relief, a little less than 12 microns, was obtained with the grating with a spatial frequency of 10 L/mm. Overall the gratings made with a film thickness of 30 microns displayed a higher relief than the gratings made with 50 microns thick films. The grating with a 4 L/mm spatial frequency, combined with 50 microns thick films, produced more pronounced reliefs than the grating with 10 L/mm.

**Figure 9:**
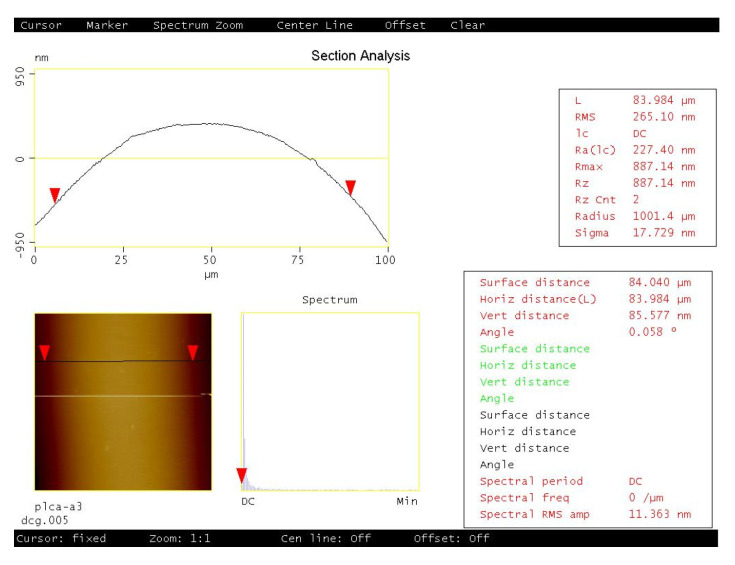
AFM analysis of the profile of one crest of a 6 L/mm grating.

The crests of the plots in Figure 3, Figure 4, Figure 5, Figure 6 and Figure 7 show a positive concavity. One of these crests was studied with an atomic force microscope, as shown in Figure 9. In addition to the concavity, the limited roughness of the surface can be appreciated.

### 3.5. Ultraviolet Light at Exposure Time

**Figure 10:**
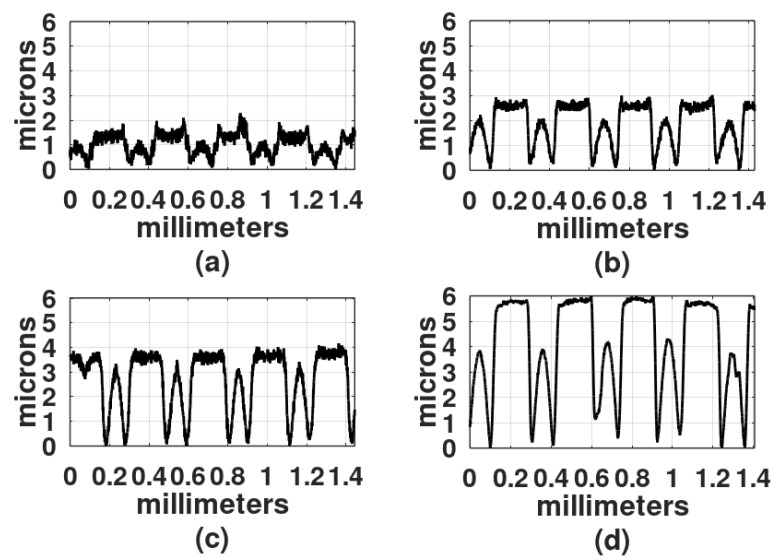
Profiles of the gratings made with UV light. Exposure times: (**a**) 1 min, (**b**) 2 min, (**c**) 4 min, and (**d**) 8 min. Spatial frequency: 6 L/mm.

It is known that DCG is more sensitive to long-UV light than to visible light [2,3] therefore, a UV mercury metal-halide pressure lamp was used in the recording. In the following set of experiments, the light source was an extended one, as shown in Figure 2b. The distance between the lamp and the recording area was 25 cm. The Ronchi grating had a spatial frequency of 6 L/mm. DCG films were 30 microns thick. Exposure times were 1 min, 2 min, 4 min, and 8 min. The development process included 10 min in water. Details of the surface profiles of the gratings are shown in Figure 10. It can be observed that the profiles show rather flat valleys and between them the crests are slightly shallower. The flat parts and the crests are a result of the clear and the opaque strips in the Ronchi gratings, respectively.

**Figure 11:**
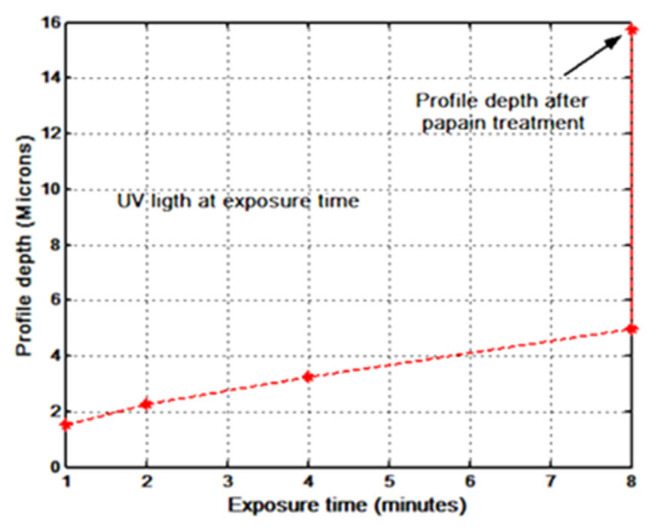
Gratings’ profile depth, as a function of exposure times to UV light. DCG films’ thickness was 30 µm.

Three of the highest reliefs of each plot in Figure 10 were measured and the mean of the three was calculated and plotted in Figure 11; the highest relief obtained was about 5 microns. For comparison, in this plot, the relief depth of the grating made with an exposure time of 8 min but developed with papain is also shown. The details of these experiments are provided in Section 3.6.

Figure 4c represents the recording with blue light and Figure 10d represents the recording with UV light. A Ronchi grating of 6 L/mm was used in both experiments. The curve in Figure 4c shows a series of peaks, with flat, rough surfaces in between. As mentioned, the flat surfaces were formed in those places where light was allowed to illuminate. Peaks are about 3 µm higher than the flat surfaces, with the width of the peaks being about the width of the flat surfaces. The curve in Figure 10d displays a series of rounded peaks between flat surfaces. In this case, the flat surfaces are 2 µm higher than the peaks and they are wider than the rounded peaks. This result suggests that hardening with blue light generates a different product compared to hardening with UV light. There is ongoing research to determine the causes of this phenomenon.

### 3.6. The Papain in the Development Process

**Figure 12:**
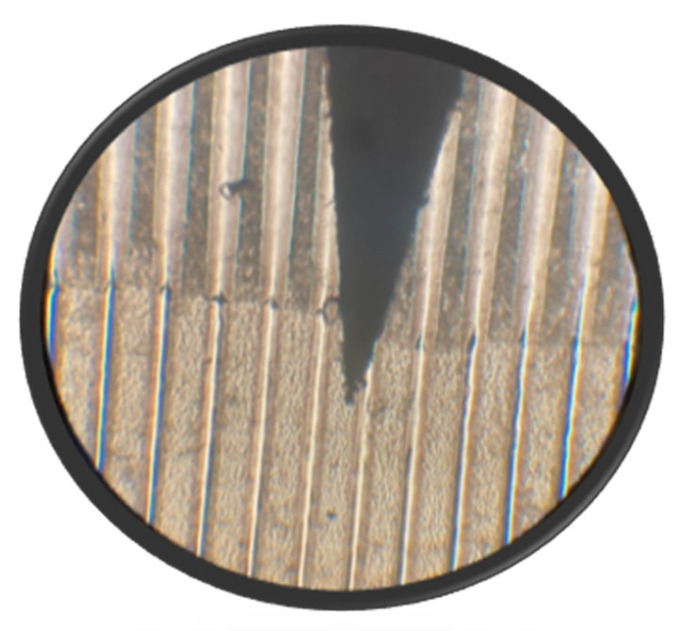
Photograph showing a grating (6 L/mm). In the upper part the development was made with papain/water. In the lower part, just water was used. The black arrowhead is pointing to one of the zones corresponding to a transparent slit in the Ronchi grating.

Although the development process with water gave good results, we explored the possibility of generating even deeper grating profiles for increased optical path difference. Thus, an alternative development process was carried out with papain [26,31,32]. Figure 12 shows a grating that was developed with both water and papain in its upper part and with just water in its lower part. 

**Figure 13:**
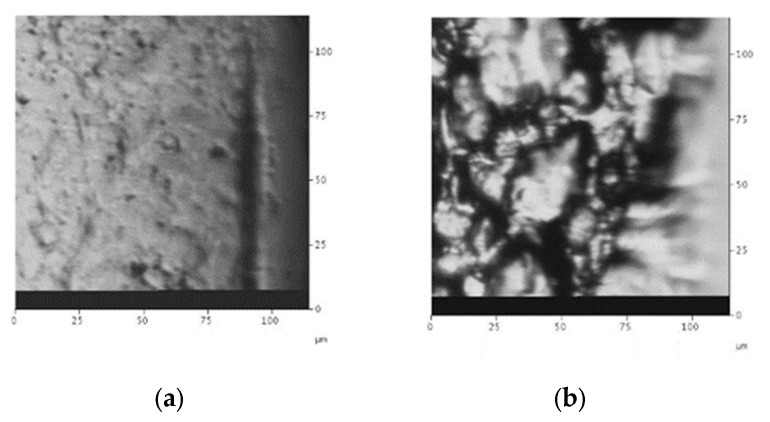
AFM photographs show part of a grating that underwent development with (**a**) just water and (**b**) with water and papain. The use of papain causes the grainy texture shown in the second photograph.

**Figure 14:**
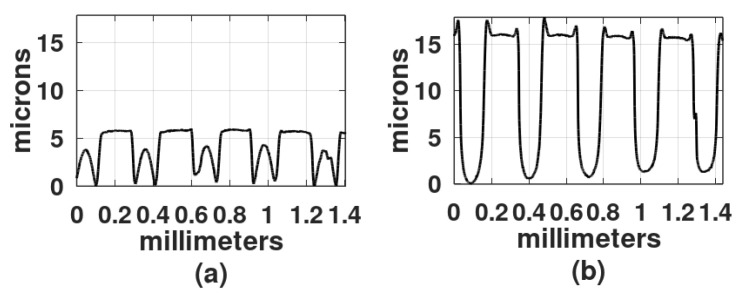
Plot of a grating that (**a**) underwent water development and later (**b**) underwent papain/water development. The depth of this last plot has increased and the crests between the flat surfaces disappeared. The relief depth value of this last graph has been plotted in Figure 11.

The action of the enzyme on gratings made with blue light is shown in Figure 13. The part of the grating that was developed with just water, Figure 13a, shows a smoother surface than the part shown in Figure 13b, which was soaked in the water/papain resulting in a very rough surface that generates scattering. The roughness seen in Figure 13b could be caused by the presence of “grains” of papain in the solution. To avoid this effect, a new mixture was made and filtered with a filter paper. The grating was then immersed in this new mixture, with a consequent reduction in surface roughness. A surface profile of the unprocessed and processed gratings (with the filtered papain mixture) can be seen in the plots of Figure 14a,b. Note that, in comparison to Figure 14a, in Figure 14b the surface profile depth is increased and the crests are no longer present between the flat parts. The relief depth is plotted in Figure 11.

### 3.7. Diffracted Orders

**Figure 15:**
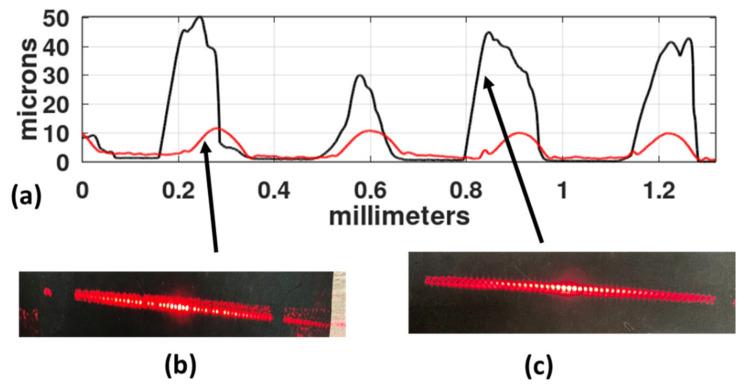
(**a**) The grating profile that shows low relief (red curve) was developed with just water. Its diffracted orders are shown in (**b**). (**c**) shows the diffracted orders that were generated by the grating with the highest profile (blue curve) in (**a**).

The action of the papain mixture was analyzed in relation to the behavior of the diffracted orders. One grating was made with a slit width of 166 microns and developed by washing it in water for 10 min. In Figure 15a, the red curve shows its profile which is characterized by shallow valleys. When illuminated with light from a He-Ne laser, the grating generated the diffracted orders shown in Figure 15b. Besides the central orders (−3, −2, −1, 0, +1, +2, +3), more orders can be seen. Following this, a different part of the grating was developed with a filtered mixture of papain and water. Its profile is shown in Figure 15a as the blue curve. The height of the peaks generated with water reach a maximum of about 7 µm, while the height of the peaks obtained with the use of filtered papain reach a maximum of 50 µm. The clean, rounded spots of the diffracted orders of the papain-digested grating can be appreciated in Figure 15c.

## 4. Discussion

The surface relief studies reported in references [14,15,16,17,18] were performed with interference patterns with spatial frequencies between 60 L/mm and 200 L/mm. At recording time, they used visible light with a wavelength of 440 nm. The developing process was performed with water and the plates were dried afterward. Irradiation of the plates with UV light with a wavelength between 250 nm and 270 nm was then performed, and the plates suffered another water development. Reliefs between 0.4 microns and 1.6 microns were obtained. Diffractive elements that work with visible light should present reliefs with less than 1 µm, thus the reported reliefs are useful because they could give good diffraction efficiencies. The process that we report in this work is different because we used UV light at the recording time, with a wavelength of 385 nm. A light with this wavelength is capable of breaking the crosslinks between the gelatin molecules, while the light used in reference [18] degrades the gelatin.

The spatial frequencies used in this article are between 4 L/mm and 10 L/mm. The reliefs measured are in the order of few microns to 50 microns. These reliefs are useful if the optical elements are meant to work with infrared light (λ = 1 µm to 15 µm). In additiion, DCG plates with high reliefs could be used to host liquid crystals.

As pointed out in Section 2 (theory), the only reference to date that has showed the use of a proteolitic enzyme in the development of DCG films is [30]. They developed an introductory test and mentioned some commentaries of preliminary experiments and applications. A deep and systematic study of the process needs to be performed. However, in this report we were able to generate quantitative results, considering variables such as film thickness, gratings spatial frequencies, wavelength of light sources, exposure times, and development with papain. Our process provides better depth reliefs; however, we have also shown that the action of the enzyme is not uniform at a microscopic scale, in the present conditions. New studies are needed to determine how to control the action of papain on gelatin and to generate better surfaces with less roughness and lower depths. In particular, the response of the enzyme needs to be improved when gratings spatial frequencies are high.

## References

[1] Kosar J. (1965). Light sensitive systems. Chemistry and Applications of Nonsilver Halide Photographic Processes.

[2] Shankoff T.A. (1968). Phase holograms in dichromated gelatin. Appl. Opt..

[3] Brandes R.G., Francois E.E., Shankoff T.A. (1969). Preparation of dichromated gelatin films for holography. Appl. Opt..

[4] Calixto S. (2003). Albumen as relief recording media for spatial distributions of infrared radiation. Fabrication of interference gratings and microlenses. Appl. Opt..

[5] Kotakonda P., Naydenova I., Martin S., Toal V. (2006). Photoinduced Surface relief studies in an acrylamide-based photopolymer. J. Op. A Pure Appl. Opt..

[6] Calixto S., Salazar M., Servin M. (1995). photosensitive element for a novel infrared to visible image converter. Appl. Opt..

[7] Calixto S. (2002). Silicone microlenses and interference gratings. Appl. Opt..

[8] Calixto S. (2007). Relief gratings and microlenses fabricated with silicone. Appl. Opt..

[9] Calixto S. (1988). Infrared recording with gelatin films. Appl. Opt..

[10] Calixto S., Andres M.V. (2015). Water vapor sensors based on the swelling of relief gratings. Adv. Mater. Sci. Eng..

[11] Calixto S., Croutxe-Barghon C., Lougnot D.J. (1999). Ultravioelt-self generating relief micro-optical elements through cross linking photopolimerazation of liquid resins. Eur. Phys. J. Appl. Phys..

[12] Navarrete E., Calixto S. (1998). Surface relief zone plates fabricated with photographic emulsions. Appl. Opt..

[13] Navarrete E., Calixto S. (2003). Continuous surface relief micro-optical elements fabricated with photographic emulsions by use of binary and half tone masks. Opt. Mater..

[14] Gulyaev S.N., Igor I. Phenomenon of period-doubling in holographic structures exposed to UV radiation. Proceedings of the Fourth International Workshop on Nondestructive Testing and Computer Simulations in Science and Engineering, SPIE, 4348.

[15] Gulyaev S.V., Ratushnyl V.P. (2003). Properties of relief-phase holograms produced by processing photographic plates with short-wavelength UV radiation and with two-stage bleaching. J. Opt. Technol..

[16] Ganzherly N.M., Gulyaev S.V., Maurer I.A. (2016). The effect of UV radiation on the properties of diffraction gratings based on dichromated gelatin. Tech. Phys. Lett..

[17] Ganzherly N.M., Gulyaev S.V., Maurer I.A. (2017). Properties of holographic structures on dichromated gelatin subjected to ultraviolet radiation. J. Opt. Technol..

[18] Ganzherly N.M., Gulyaev S.N., Maurer I.A. (2018). The effect of short-wave UV radiation in recording holographic structures on gelatin-containing recording media (overview). Opt. Spectrosc..

[19] Sio L.D., Cuennet J.C., Vasdekis A., Psaltis D. (2010). All-optical switching in an optofluidic polydimethylsiloxane: Liquid crystal grating defined by cast-molding. Appl. Phys. Lett..

[20] Sio L.D., Vasdekis A., Guennet J.G., Luca A.D., Pane A., Psaltis D. (2011). Silicon oxide deposition for enhanced optical switching in polydimethylsiloxane liquid crystal hybrids. Opt. Express.

[21] Rochon P., Batalla E., Natansohn A. (1995). Optically induced gratings on azoaromatic polymer films. Appl. Phys. Lett..

[22] Kim D.Y., Tripathy S.K., Li L., Kumar J. (1995). Laser-induced holographic surface relief gratings on nonlinear optical films. Appl. Phys. Lett..

[23] Lagugne Labarthet F., Rochon P., Natansohn A. (1999). Polarization analysis of diffracted orders from a birefringence grating recorded on azobenzene containing polymer. Appl. Phys. Lett..

[24] Park J.-H., Yu C.-J., Kim J., Chung S.-J., Lee S.-D. (2003). Concept of a liquid-crystal polarization beamsplitter based on binary phase gratings. Appl. Phys. Lett..

[25] Calixto S., Garcia-Cordero J.L., Cedillo-Alcantar D.F., Naydenova I., Garnica G. (2020). Birefringent optofluidic gratings. Opt. Express.

[26] Piroda L., Moriconi M. (1988). An effective processing agent for dichromated gelatin. Opt. Commun..

[27] Carrol B.H., Higgins G.C., James T.H. (1980). Introduction to Photographic Theory. The Silver Halide Process.

[28] Calixto S., Ganzerly N.M., Gulyaev S.V., Figueroa-Gerstenmier S. (2018). Gelatin as photosensitive material. Molecules.

[29] Smith H.M. (1968). Photographic relief images. J. Opt. Soc. Am..

[30] Sosnowsky T.P., Kogelnik H. (1970). Ultraviolet hologram recording in dichromated gelatin. Appl. Opt..

[31] Lowe G. (1970). The structure and mechanism of action of papain, Philosophical transactions of the Royal society of London. Phil. Trans. R. Soc. Lond. B Biol. Sci..

[32] Homaci A.A., Sajedi R.H., Sariri R., Seyfzadeh S., Stevanato R. (2010). Cysteine enhances activity and stability of immobilized papain. Aminoacids.

[33] Output Spectrum of a Typical Mercury Metal-Halide Lamp Shows Peaks at 385nm, 422nm, 497nm, 540nm, 564nm, 583nm (Highest), 630nm, and 674nm. https://upload.wikimedia.org/wikipedia/en/9/93/Metal_Halide_Lamp_Spectrum.jpg..

